# An Underactuated Omnidirectional Docking Mechanism for Modular Serpentine Robots with DNA-Inspired Helical Continuum Units

**DOI:** 10.3390/biomimetics11070506

**Published:** 2026-07-18

**Authors:** Yiqi Zhang, Tuo Zhang, Gengbiao Chen, Lairong Yin, Amin Ye

**Affiliations:** 1 College of Mechanical and Vehicle Engineering, Changsha University of Science and Technology, Changsha 410114, China; 202403010306@csust.edu.cn (Y.Z.); 23203030757@csust.edu.cn (T.Z.); chengengbiao@csust.edu.cn (G.C.); 2 Key Laboratory of Safety Design and Reliability Technology for Engineering Vehicle, Changsha 410114, China; 3 Disaster Prevention and Rescue College, Hunan Vocational Institute of Safety Technology, Changsha 410151, China; yeamin@stu.csust.edu.cn

**Keywords:** serpentine robot, continuum robot, underactuated gripper, robot docking, adaptive control, mechanical modeling

## Abstract

Bio-inspired serpentine robots show strong potential for operation in unstructured environments, yet existing systems often lack reliable modular docking, adaptive grasping, and an effective balance between structural stiffness and motion dexterity. This study proposes a Modular Omnidirectional Serpentine Robot (MOSR) that integrates a DNA-inspired tendon-driven helical continuum unit, an underactuated omnidirectional spherical docking gripper, and adaptive gripper fingers within a single module. The helical continuum unit provides two-degree-of-freedom compliant bending while improving axial stiffness through interleaved helices and a central constraint structure. The spherical docking gripper adopts a linkage-spring-slider underactuated mechanism to accommodate effective-diameter variations and support stable one-to-one and one-to-many docking. Gripper kinematics are modeled using an improved Denavit–Hartenberg method, and the workspace is verified by MATLAB simulation. Equivalent torsional and bending stiffness models are established for the helical continuum unit and validated by finite element analysis, with mean relative errors of 11.57% and 17.95%, respectively. Docking-angle analysis based on the receiver polar angle (*θ_rec_*) and engager azimuth angle (*θ_eng_*) shows that 61.1% of the receiver surface lies within the feasible docking region at an opening distance of 5.7 mm. A 3D-printed Polyamide 1010 prototype achieves a locomotion speed of 15.3 mm/s on grass and demonstrates terrain traversal, planar steering, obstacle crossing, adaptive grasping, and stable straight and oblique docking. These results verify the feasibility of integrating locomotion, grasping, and modular reconfiguration within a single serpentine robot module.

## 1. Introduction

Bio-inspired serpentine robots have emerged as promising candidates for versatile tasks in unstructured environments, such as confined-space exploration, minimally invasive surgery, and disaster rescue, due to their inherent compliance, dexterous motion, and ability to adapt to curvilinear paths [1,2,3]. The integration of modular design and multifunctional manipulation further expands their application potential, as modular self-reconfigurable robots can adjust morphologies to meet diverse task requirements [4]. However, three core technical demands remain unmet to realize their practical deployment: first, the need for omnidirectional, stable, and scalable modular connectivity that supports both one-to-one and one-to-many docking; second, adaptive grasping capabilities for objects of varying shapes and sizes without complex control systems; and third, a balanced trade-off between structural stiffness (for load-bearing) and motion dexterity (for environmental adaptation) in continuum units [5]. Recent work has advanced continuum-robot kinematic analysis and intelligent mobile robot design [6,7]. Parallel progress has also been reported in vision-guided robotic grasping and bio-inspired compliant mechanisms for robotic applications [8,9]. These developments indicate a growing trend toward integrating structural design, motion analysis, perception, and functional adaptability in robotic systems, which further motivates the present study of a modular serpentine robot with combined locomotion, grasping, and reconfiguration capabilities.

Despite progress in soft robotics, critical gaps remain in meeting the three demands outlined above. Regarding modular connectivity, most existing designs are unidirectional or restricted to one-to-one docking. They lack adaptability to diameter variations caused by shell thickness, which often leads to collisions between module bases and necks [10]. Moreover, conventional docking mechanisms—even those claiming universal compatibility—struggle with lightweight modular robots due to insufficient momentum for reliable engagement, while cross-platform standard incompatibilities further limit reconfigurability [11]. Regarding adaptive grasping, most underactuated soft grippers lack effective mechanisms to counteract torque and shear forces during contact, resulting in unstable grasping of irregular objects [12,13]. Although some designs incorporate multi-mode grasping, their contact area and docking angle selectivity remain limited, and friction-related problems under dry or wet conditions further degrade performance [14]. Regarding continuum unit design, pneumatically actuated systems require bulky pumps and hoses, hindering untethered operation and miniaturization [15]; tendon-driven structures often suffer from wire slack during deflection and fail to balance stiffness with workspace [16]. Additionally, the inherent nonlinearity and shape instability of soft materials impose challenges for precise control under external forces [17].

To address these interconnected gaps, this study proposes an integrated design for modular bio-inspired serpentine robots with three core innovations, each directly targeting one of the above limitations. First, to overcome the stiffness–dexterity trade-off in continuum units, we develop a tendon-driven helical continuum unit inspired by the DNA double helix. Building on prior DNA-inspired wire-routing concepts [18] but incorporating interleaved helices and constraint structures (mimicking DNA base pairs), this design enhances axial stiffness while maintaining two degrees of freedom (DOF) for compliant motion, thus overcoming the trade-off that plagues conventional tendon-driven continuum robots [19]. Second, to resolve the limitations of unidirectional and non-adaptive docking, we propose an omnidirectional docking mechanism based on a spherical gripper. Adopting the “universal compatibility” philosophy of advanced docking systems [11] but integrating a linkage-spring-slider underactuated structure, it adapts to diameter differences. With a 40% finger-width ratio and a top circular hole, this mechanism expands the contact area and the range of feasible docking angles, enabling stable one-to-one and one-to-many dockings. Third, to remedy the lack of torque/shear-force neutralization in grasping, we design underactuated gripper fingers with a passive adaptive enveloping mechanism. Improving upon existing underactuated grasping principles [14] by incorporating linkages, spring-loaded telescopic rods, and contact plates, this design effectively counteracts torque and shear forces, ensuring reliable grasping of diverse objects.

Based on the above innovations, the primary objectives of this study are to (1) establish a kinematic model for the proposed gripper using an improved Denavit–Hartenberg (D-H) method and verify its workspace; (2) derive and validate equivalent torsional and bending stiffness models for the helical continuum unit via finite element analysis (FEA); (3) characterize the docking performance (feasible angle range, stability) of the omnidirectional docking mechanism; and (4) demonstrate the locomotion, grasping, and reconfiguration capabilities of a 3D-printed prototype in practical scenarios [20].

The remainder of this paper is structured as follows: Section 2 details the overall design of the modular serpentine robot, including the underactuated omnidirectional docking mechanism, the helical continuum unit, and the electronic and communication system. Section 3 presents the kinematic and stiffness modeling of the gripper and the helical continuum unit, along with simulation results. Section 4 describes the experimental setup for prototype validation. Section 5 reports the experimental results, including stiffness validation, kinematic validation, grasping performance, docking performance, and locomotion tests. Section 6 discusses the implications and limitations of the findings. Finally, Section 7 concludes the paper with a summary of key contributions and future research directions.

## 2. Structural Design of the MOSR

**Figure 1:**
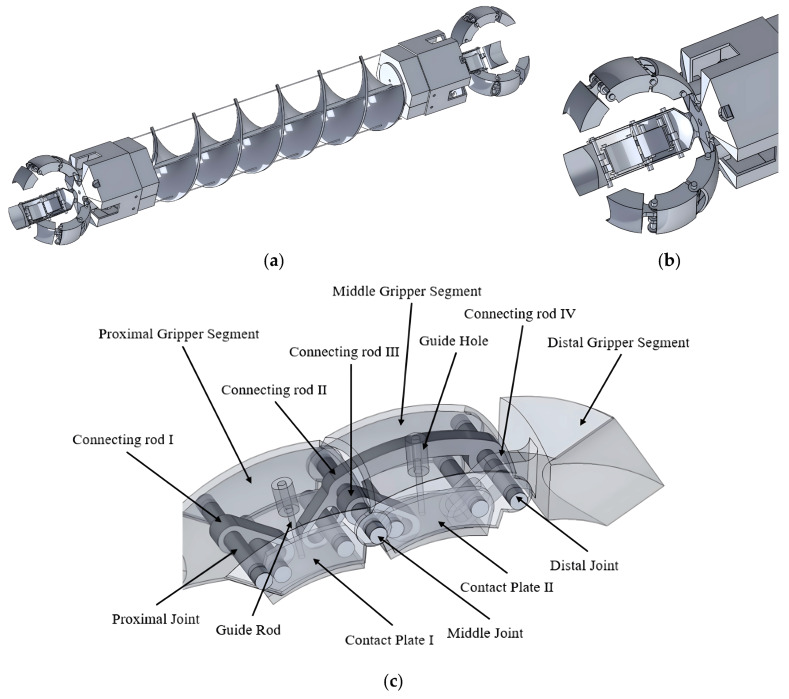
Structural design of the MOSR: (**a**) overall structure of the MOSR; (**b**) spherical docking gripper; (**c**) underactuated finger.

The proposed MOSR comprises a helical continuum unit and two spherical docking grippers, as shown in Figure 1a.

The MOSR is designed to provide each module with high kinematic flexibility, omnidirectional docking capability, and integrated locomotion and manipulation. The design objectives are threefold: (1) to endow a single module with high motion flexibility; (2) to enable both one-to-one and one-to-many omnidirectional docking, thereby supporting diverse locomotion configurations; and (3) to integrate locomotion and manipulation within a single module. The tendon-driven continuum unit provides two degrees of freedom for bending to enable compliant motion, while the spherical docking grippers enable both intermodular docking and object manipulation.

### 2.1. Design of the Underactuated Omnidirectional Docking Mechanism

#### 2.1.1. Grasping–Docking Morphing Mechanism

We designed a novel grasping–docking mechanism that combines adaptive grasping with modular docking, as shown in Figure 1b. The mechanism includes three structurally symmetric underactuated fingers arranged circumferentially at 120° intervals. Uniform gaps between adjacent fingers provide enough space for independent motion and prevent mechanical interference. As the fingers close, the three fingers retract inward along predefined trajectories. Their outer contours change from a discrete claw-like shape to an approximately spherical surface and finally form a regular spherical profile. Another identical spherical docking gripper can securely grasp this spherical profile. In this way, two grippers achieve stable intermodular docking and form an integrated mechanism with omnidirectional docking capability.

To accommodate the effective diameter variation caused by the shell thickness of the spherical docking gripper, we developed an underactuated finger based on a linkage-spring-slider architecture. The linkage transmits motion to the slider. The spring supplies an adaptive preload and allows the finger to adjust its closing displacement after contacting the spherical surface. This design enables stable grasping of spherical structures with different effective diameters. The underactuated configuration also supports passive adaptive closure without complex control. It improves the versatility and reliability of the mechanism and keeps the structure simple and lightweight. This design offers an effective mechanical solution for modular spherical grasping and omnidirectional docking.

#### 2.1.2. Design of the Underactuated Finger

To achieve stable grasping of objects with different sizes and shapes, we developed a novel transmission mechanism for the fingers of the spherical docking gripper, as shown in Figure 1c. The mechanism consists of linkages, a spring-loaded telescopic rod, contact plates, and a coupled slider moving along a guide rail. It passively conforms to object surfaces and produces adaptive enveloping. This feature compensates for diameter variations caused by the spherical shell thickness and also allows the gripper to capture irregular objects.

In the overall structure, Links I, II, III, and IV connect to internal Contact Plates I and II through guide rails. As the contact plates move along the rails, Links I and II rotate about their joints. Spring-loaded telescopic rods connect the contact plates to the upper part of the finger shell. These rods resist the torque and shear forces acting on the contact plates and improve grasping stability.

When Contact Plate I first touches the object surface, it moves along the outer shell. This motion displaces the pin inside the slider and drives Link II to rotate. When Contact Plate II touches the object, Link IV rotates and brings the fingertip into contact with the object surface. These motions create a passive adaptive enveloping grasping mechanism. The finger then conforms automatically to the target geometry and achieves stable and efficient grasping.

The circular opening at the top of the gripper enlarges the docking region. Each finger occupies 40° of the 360° central planes of the gripper. This design maintains sufficient contact area and supports multi-angle modular docking. It also improves the flexibility and scalability of the spherical docking gripper in omnidirectional reconfiguration and cooperative manipulation.

### 2.2. Structural Design of the Helical Continuum Unit

As shown in Figure 1a, inspired by the double-helical architecture of DNA and the constraint mechanism of base pairs, we designed a helical continuum unit that combines interlaced helical chains with a central constraint structure. This design achieves two degrees of freedom in bending and markedly enhances axial stiffness. The unit consists of a helical skeleton printed with Polyamide 1010 and four pairs of driving tendons routed through the skeleton. The helical skeleton includes two interlaced helices and a slender constraint shaft. The slender shaft mimics the role of base pairs in DNA, restricts excessive deformation of the helical chains, and improves the overall stiffness and structural stability.

In the design of continuum units, bending capability and load-carrying capacity present an inherent trade-off. Under a fixed bending angle, a higher load capacity requires greater tendon tension. The continuum structure must therefore provide sufficient axial stiffness to resist this tension. Conventional single-chain continuum structures, such as slotted flexible rods, can achieve two degrees of freedom of bending, but their axial stiffness decreases significantly as the bending angle increases. Under high tendon tension, these structures tend to deform unstably. Multi-chamber pneumatic continua can regulate stiffness, but they require complex pneumatic control and do not suit the rapid reconfiguration demanded by modular units. For this reason, we adopted a tendon-driven scheme. This scheme offers a compact structure, low weight, and simple control. It does not rely on an external fluid supply. It also facilitates modular integration and multi-unit assembly. These features make it well-suited for space-constrained flexible mobile systems such as serpentine robots.

### 2.3. Electronic Control and Communication System Design

**Figure 2:**
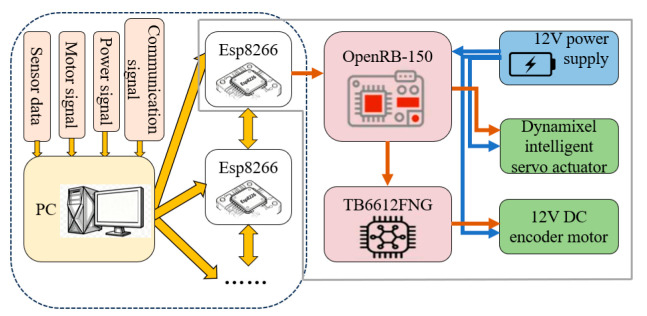
Electronic control and communication system.

As shown in Figure 2, the OpenRB-150 microcontroller precisely controls the Dynamixel servo motor through the TB6612FNG driver and actuates tendon extension and retraction in the helical continuum unit, which generates two-degree-of-freedom bending. The ESP8266 module supports wireless communication between modules and ensures real-time signal transmission and coordinated control during one-to-one and one-to-many docking of the omnidirectional docking mechanism. A custom printed circuit board integrates the microcontroller, Wi-Fi communication module, and motor driver into a compact electronic architecture. This design meets the requirements of the MOSR for miniaturization and high system integration.

## 3. Modeling and Analysis of the MOSR

This section presents the modeling and performance characterization of the proposed helical continuum mechanism. We first derive the equivalent torsional stiffness and equivalent bending stiffness of the continuum joint. We then combine the theoretical model with finite element analysis to evaluate its stiffness characteristics quantitatively. After that, we analyze the kinematic behavior and docking performance of the spherical connector. These results provide a theoretical basis for the design and optimization of the modular mechanism.

### 3.1. Kinematic Modeling and Workspace Analysis of the Gripper

#### 3.1.1. Kinematic Modeling of the Joint Actuator Based on the D-H Method

The Denavit–Hartenberg (D-H) method is widely used in kinematic modeling of linkage mechanisms and articulated systems. It places no special restriction on link geometry or joint rotation form, so it can describe, in principle, any mechanical system composed of links and joints. To establish the kinematic model of the finger joint actuator, we define a reference coordinate frame for each phalanx.

In D-H modeling, each coordinate frame consists of three orthogonal axes, *X*, *Y*, and *Z*. The *Z*-axis coincides with the joint axis of motion. The *X*-axis describes the relative position between two adjacent coordinate frames. The *Y*-axis follows the right-hand rule.

The standard D-H parameterization uses four parameters, *a_i_*, *d_i_*, *α_i_* and *θ_i_* to describe the pose of the *i-th* link relative to the previous link. Here, ai denotes the length of link *L_i_*; *d_i_* denotes the displacement along the *Z_i_*_−__1_-axis; *α_i_* denotes the twist angle of link *L_i_* relative to the previous link; *θ_i_* represents the joint angle about the *Z_i_*_−__1_-axis.

These parameters define the homogeneous transformation matrix *A_i_* between adjacent coordinate frames.

(1) $$A_{i}=\left[\begin{array}{cccc} \cos\theta_{i} & -\sin\theta_{i}\cos\alpha_{i} & \sin\theta_{i}\sin\alpha_{i} & a_{i}\cos\theta_{i}\\ \sin\theta_{i} & \cos\theta_{i}\cos\alpha_{i} & -\cos\theta_{i}\sin\alpha_{i} & a_{i}\sin\theta_{i}\\ 0 & \sin\alpha_{i} & \cos\alpha_{i} & d_{i}\\ 0 & 0 & 0 & 1 \end{array}\right]$$

The overall transformation matrix of the end-effector with respect to the base coordinate frame can be expressed as

(2) $$T=A_{1}A_{2}\cdots A_{n}$$

where *n* denotes the number of joints.

#### 3.1.2. Workspace Modeling and Simulation of the Finger

Based on the homogeneous transformation relationship, the position of an arbitrary point P on the fingertip in the base coordinate frame can be expressed as

(3) $$P_{i}=TP_{0}$$

where Pi denotes the coordinates of point *P* in the *x_i_*, *y_i_*, *z_i_* coordinate frame, and *P_0_* denotes its initial coordinates in the end-effector coordinate frame. By specifying the range of motion of each joint and performing numerical simulations in MATLAB (Version Number: R2025b), we can obtain the trajectory and spatial distribution of this point, which determine the workspace of the finger.

To reduce modeling complexity, we simplified the finger structure while preserving its kinematic characteristics. For continuum or flexible joints, we used a discretization approach and approximated them as a finite number of rigid segments. We then established the kinematic model using a modified D-H method. Let the coordinates of point *P* at the fingertip in the Cartesian coordinate system be (*P_x_*, *P_y_*, *P_z_*). The homogeneous transformation matrix *A_i_* associated with the *i-th* coordinate frame can be written as

(4) Ai=Tii−1=cosθi−sinθi0ai-1cosθisinθicosθi0ai-1sinθi00100001

where a denotes the link length, *θ* denotes the link contraction angle, and *i* denotes the *i-th* coordinate frame. The relationship between the coordinates of point *P* and the transformation matrix Ai can then be expressed as

(5) T40=A1⋅A2⋅A3⋅A4

(6) T40=cos(θ1+θ2+θ3)−sin(θ1+θ2+θ3)0Pxsin(θ1+θ2+θ3)cos(θ1+θ2+θ3)0Py001Pz0001

(7) $$\left\{\begin{array}{c} P_{x}=a_{1}\cos\theta_{1}+a_{2}\cos(\theta_{1}+\theta_{2})+a_{3}\cos(\theta_{1}+\theta_{2}+\theta_{3})\\ P_{y}=a_{1}\sin\theta_{1}+a_{2}\sin(\theta_{1}+\theta_{2})+a_{3}\sin(\theta_{1}+\theta_{2}+\theta_{3})\\ P_{z}=0 \end{array}\right.$$

By specifying the range of variation for each joint angle and performing numerical simulations in the MATLAB Robotics Toolbox, we can compute the trajectory and spatial distribution of point *P*, and thus determine the workspace of the finger. The simulation results help evaluate the range of motion, dexterity, and spatial reachability of the finger, and provide a theoretical basis for subsequent control design.

### 3.2. Dynamic Modeling of Underactuated Gripper

**Figure 3:**
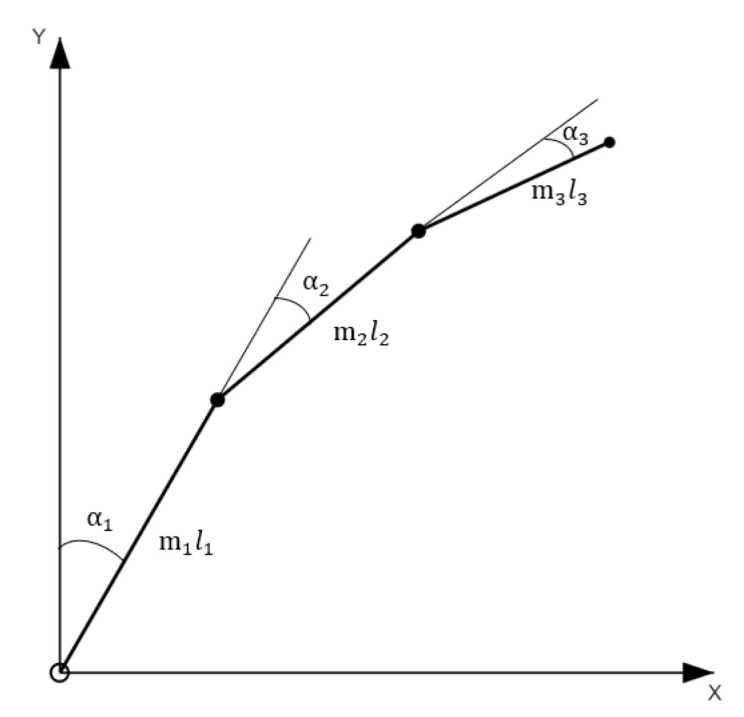
Three-link model.

Considering the complexity of the equations for a multi-degree-of-freedom system, the gripper is treated as a three-link model. The Lagrange method is used to analyze the dynamic equations of this model, as shown in Figure 3.

In the diagram, parameter $m_{i}$ represents the simplified mass of the link after the finger joint; $\alpha_{i}$ represents the rotation angle of the i-th link joint; and $l_{i}$ represents the length of the i-th link (*i* = 1, 2, 3).

Considering the structural characteristics of the gripper and for computational simplicity, the center of mass of link 1 is equivalent to the center of the link, and the centers of mass of links 2 and 3 are located at the ends of the links. Therefore, the moments of inertia of each link in the gripper are expressed as

(8) $$\left\{\begin{array}{c} J_{1}=\frac{m_{1}l_{1}^{2}}{12}\\ J_{2}=\frac{m_{2}l_{3}^{2}}{3}\\ J_{3}=\frac{m_{3}l_{3}^{2}}{3} \end{array}\right.$$

The center of gravity of each link is represented as follows:

(9) $$W_{1}=\left[\begin{array}{c} x_{1}\\ y_{1} \end{array}\right]=\left[\begin{array}{c} \frac{1}{2}l_{1}s_{1}\\ \frac{1}{2}l_{1}c_{1} \end{array}\right]$$

In the formula: $s_{1}$ represents $\sin\alpha_{1}$; $c_{1}$ represents $\cos\alpha_{1}$.

(10) $$W_{2}=\left[\begin{array}{c} x_{2}\\ y_{2} \end{array}\right]=\left[\begin{array}{c} l_{1}s_{1}+l_{2}s_{12}\\ l_{1}c_{1}+l_{2}c_{12} \end{array}\right]$$

In the formula: $s_{12}$ represents $\sin\left(\alpha_{1}+\alpha_{2}\right)$; $c_{12}$ represents $\cos\left(\alpha_{1}+\alpha_{2}\right)$.

(11) $$W_{3}=\left[\begin{array}{c} x_{3}\\ y_{3} \end{array}\right]=\left[\begin{array}{c} l_{1}s_{1}+l_{2}s_{12}+l_{2}s_{123}\\ l_{1}c_{1}+l_{2}c_{12}+l_{2}c_{123} \end{array}\right]$$

In the formula: $s_{123}$ represents $\sin\left(\alpha_{1}+\alpha_{2}+\alpha_{3}\right)$; $c_{12}$ represents $\cos\left(\alpha_{1}+\alpha_{2}+\alpha_{3}\right)$.

The link velocity $v_{i}$ is expressed as

(12) $$v_{i}=\sqrt{{\dot{x}}_{i}^{2}+{\dot{y}}_{i}^{2}}$$

The angular velocity of the link, $\omega_{i}$ is expressed as

(13) $$\left\{\begin{array}{c} \omega_{1}={\dot{\alpha }}_{1}\\ \omega_{2}={\dot{\alpha }}_{1}+{\dot{\alpha }}_{2}\\ \omega_{3}={\dot{\alpha }}_{1}+{\dot{\alpha }}_{2}+{\dot{\alpha }}_{3} \end{array}\right.$$

The kinetic energies of each link are as follows:

(14) $$\left\{\begin{array}{c} K_{1}=\frac{1}{2}m_{1}v_{1}^{2}+\frac{1}{2}J_{1}\omega_{1}^{2}\\ K_{2}=\frac{1}{2}m_{2}v_{2}^{2}+\frac{1}{2}J_{2}\omega_{2}^{2}\\ K_{3}=\frac{1}{2}m_{3}v_{3}^{2}+\frac{l}{2}J_{3}\omega_{3}^{2} \end{array}\right.$$

The total kinetic energy of the gripper is the sum of the kinetic energies of all the links:

(15) $$K=K_{1}+K_{2}+K_{3}$$

Taking the zero point of potential energy as the origin of the base coordinate system, the potential energy *P* of each rod is

(16) $$\left\{\begin{array}{c} P_{1}=m_{1}gl_{1}c_{1}/2\\ P_{2}=m_{2}g(l_{1}c_{1}+l_{2}c_{12})\\ P_{3}=m_{3}g(l_{1}c_{1}+l_{2}c_{12}+l_{3}c_{123}) \end{array}\right.$$

The total potential energy is the sum of the potential energies of each rod:

(17) $$P=P_{1}+P_{2}+P_{3}$$

The Lagrangian function of the hand is

(18) L=K−P

Its general form is

(19) $$F_{i}=\frac{d}{dt}\left(\frac{\partial L}{\partial {\dot{q}}_{i}}\right)-\frac{\partial L}{\partial q_{i}}$$

Substituting (18) into the general Lagrange form, we get

(20) $$\tau_{i}=\frac{d}{dt}\left(\frac{\partial L}{\partial {\dot{\alpha }}_{i}}\right)-\frac{\partial L}{\partial \alpha_{i}}=\frac{d}{dt}\left(\frac{\partial K}{\partial {\dot{\alpha }}_{i}}\right)-\frac{\partial K}{\partial \alpha_{i}}+\frac{\partial P}{\partial \alpha_{i}}$$

In the formula, $\tau_{i}$ is the driving torque of the i-th joint.

Substituting Equations (8) to (18) into Equation (20) and solving it using MATLAB programming, we can obtain the relationship between the driving torque and the joint rotation angle during the movement, which is the dynamic equation of the gripper. The simplified relationship is

(21) $$A(\alpha )\ddot{\alpha }+B(\alpha ){\dot{\alpha }}^{2}+{\dot{\alpha }}^{T}C(\alpha )\dot{\alpha }+D(\alpha )=\tau$$

In the formula: τ is the driving torque of the gripper; $A\left(\alpha \right)$ is the mass matrix of the center of mass of the connecting rod; $B\left(\alpha \right)$ is the centrifugal term; $C\left(\alpha \right)$ is the Coriolis force matrix; $D\left(\alpha \right)$ is the gravity matrix.

### 3.3. Analysis of Docking Angles for the Omnidirectional Docking Mechanism

**Figure 4:**
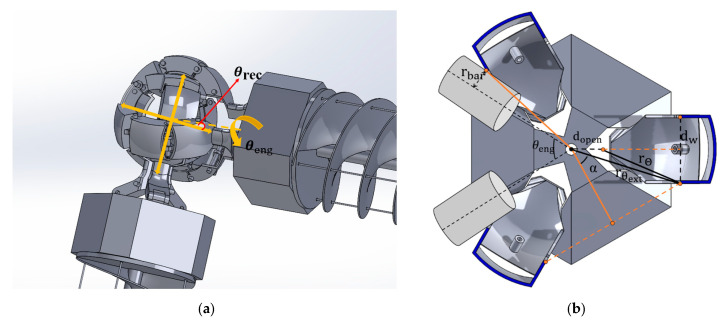
Structural design of the MOSR: (**a**) definitions of *θ_rec_* and *θ_eng_*; (**b**) geometric relationship between *θ_rec_* and *θ_eng_.*

To analyze the feasible docking angles of the proposed docking mechanism, we define the enveloping spherical docking gripper as the engager and the enveloped counterpart as the receiver. As shown in Figure 4a,b, we introduce two angular parameters to characterize the docking range of the mechanism: *θ_rec_* denotes the polar angle of the receiver docking direction, and *θ_eng_* denotes the azimuthal docking range of the engager about its *Z*-axis.

To achieve successful docking, the engager must approach the receiver at an appropriate docking angle and maintain a secure grasp throughout the docking process. Once docking is completed, the mechanism should prevent relative rotation between the receiver and the engager. To adjust the docking angle, the engager must disengage and re-engage over different ranges of *θ_rec_* and *θ_eng_* before the final engagement.

The geometric relationship between *θ_rec_* and *θ_eng_* can be calculated as

(22) $$\theta_{\mathrm{eng}}=\left\{\begin{array}{c} 2\left(\alpha -\arcsin\frac{r_{\mathrm{bar}}}{r_{\theta_{\mathrm{ext}}}}\right)\\ 2\pi \end{array}\right.$$

where

(23) $$\alpha =\frac{\pi }{3}-\arcsin\left(\frac{0.5d_{w}}{r_{\theta_{\mathrm{ext}}}}\right)$$

(24) $$r_{\theta_{\mathrm{ext}}}={\left({\left(d_{w}/2\right)}^{2}+{\left(d_{\mathrm{open}}+\sqrt{r_{\theta }^{2}-{\left(d_{w}/2\right)}^{2}}\right)}^{2}\right)}^{1/2}$$

(25) $$r_{\theta }=r\cos\theta_{rec}$$

To ensure stable and reliable docking, the engager must match the docking angle accurately and maintain stable physical contact throughout the engagement process. Angle adjustment and an accurate approach strategy play a critical role in the coordinated operation of multi-module systems. The feasibility of the docking angle depends on the workspace of the fingers, whereas the load-carrying capacity of the helical continuum unit depends on its stiffness characteristics. We therefore evaluate structural reliability through modeling and finite element analysis.

The omnidirectional docking mechanism is composed of three identical underactuated gripper fingers described in Section 3.2. Therefore, the mechanical behavior of the docking mechanism is governed by the dynamic characteristics of the underactuated gripper rather than by an independent mechanical subsystem. The dynamic model established in Section 3.2 provides the theoretical basis for adaptive contact force generation and stable engagement during the docking process, whereas the analysis presented in this section focuses on the geometric constraints and the feasible docking range required for successful omnidirectional docking.

### 3.4. Modeling of the Equivalent Torsional and Bending Stiffness of the Helical Continuum Unit

The body of the MOSR adopts a helical configuration. Its force transmission relies on the helical continuum unit, and the load-carrying capacity of the unit depends strongly on its stiffness characteristics. For stiffness analysis, we can simplify a single helical continuum unit as a mechanical spring. To establish the equivalent stiffness model of the body of the MOSR, we make the following assumptions:

(1) The helical strands are uniform and isotropic, and their stress–strain relationship is linear.

(2) We simplify the helical continuum unit of the MOSR as an elastic rod with initial twist and a rectangular cross-section.

(3) We treat the stiffness of each helical strand as equivalent to that of a simplified elastic rod.

Based on these assumptions, we can derive the equivalent torsional stiffness and equivalent bending stiffness of the helical continuum unit through mechanical modeling. We then analyze its load-carrying capacity and motion performance under practical operating conditions. The stiffness analysis of the helical continuum unit provides an important theoretical basis for evaluating its mechanical performance during the design process.

#### 3.4.1. Equivalent Torsional Stiffness of the Helical Strand

**Figure 5:**
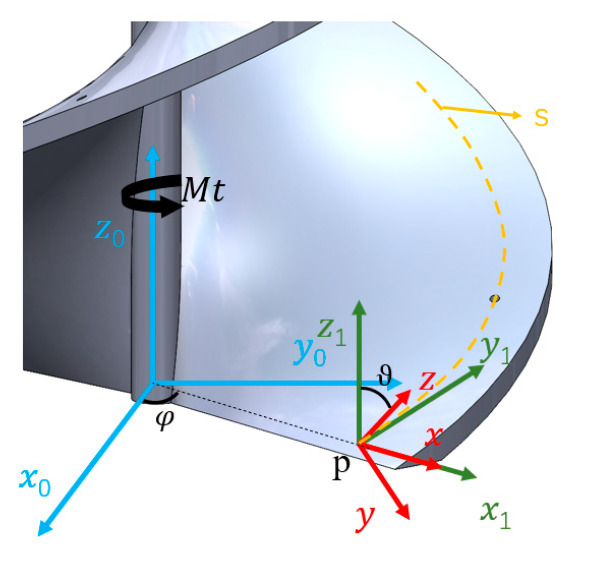
Reference frame of the helical continuum unit under torque.

Figure 5 illustrates the helical strand under an applied torsional moment. The dashed line represents the centerline of the helical strand, where dashed line *s* denotes the centroidal axis of the helical chain cross-section. The base coordinate frame $\left\{o_{0},\;x_{0},\;y_{0},\;z_{0}\right\}$ is attached to the centerline of the helical continuum unit. Let *p* be an arbitrary point on line *s*. We establish a body-fixed coordinate frame at point p as follows. (1) We translate the base coordinate frame to point *p* and rotate it about the *z*-axis by an angular variable *φ*, which gives the intermediate coordinate frame $\left\{p,x_{1},y_{1},z_{1}\right\}$. (2) We rotate $\left\{p,x_{1},y_{1},z_{1}\right\}$ about the *x_1_*-axis by an angle *ϑ* to obtain the body-fixed coordinate frame $\left\{p,x,y,z\right\}$. Note that the *z*-axis indicates the tangent direction of *s*, and the helix angle satisfies *α*$=\frac{\pi }{2}-\vartheta$. When the helical strand undergoes torsional deformation under an applied torsional moment *M_t_*, the projections of the deformation onto the *y*-axis and *z*-axis can be expressed as

(26) $$\kappa_{y}=-\overset{\cdot }{\varphi }\sin\vartheta ,\;\tau_{z}=\overset{\cdot }{\varphi }\cos\vartheta$$

The corresponding projections of the moment onto the cross-section at point *p* in the body-fixed coordinate frame can be derived as

(27) $$M_{y}=S_{y}(\kappa_{y}-\kappa_{y0}),\;M_{z}=S_{z}(\kappa_{z}-\tau_{z0})$$

where $\kappa_{y_{0}}=-\dot{\varphi }sin\vartheta_{0}$, $\tau_{z_{0}}=\dot{\varphi }cos\vartheta_{0}$ and $\vartheta_{0}=\frac{\pi }{2}-\alpha_{0}$, with *α_0_* denoting the initial helix angle of the strand. Here, *S_y_* denotes the bending stiffness of the helix about the *y*-axis, and *S_z_* denotes the torsional stiffness of the helix about the *z*-axis. Based on the definitions of bending stiffness and torsional stiffness, we obtain

(28) $$S_{y}=EIh,\;S_{z}=GJh$$

where *I_ℎ_* and *J_ℎ_* denote the moment of inertia and the second moment of area of the helix cross-section, respectively. The projection of the torsional moment *M_t_* onto the axis can then be expressed as

(29) $$M_{y}=-M_{t}\sin\vartheta ,\;M_{z}=M_{t}\cos\vartheta$$

By combining Equations (28) and (29), we obtain

(30) $$M_{t}\sin\vartheta =S_{y}(\dot{\varphi }\sin\vartheta -{\dot{\varphi }}_{0}\sin\vartheta_{0})$$

(31) $$M_{t}\cos\vartheta =S_{z}(\dot{\varphi }\cos\vartheta -{\dot{\varphi }}_{0}\cos\vartheta_{0})$$

According to [21], the above equation can be further derived as

(32) $$\dot{\varphi }-{\dot{\varphi }}_{0}=\frac{M_{t}}{S_{z}}[1+(S_{z}/S_{y}-1)\sin^{2}\vartheta_{0}]$$

Because $\dot{\varphi }=d\varphi /dz$, $dz_{0}/dz=\cos\vartheta$, we have $\varphi^{\prime }=\dot{\varphi }/\cos\vartheta$*,* Substituting this relation into Equation (32) gives

(33) $$\varphi^{\prime }-{\varphi^{\prime }}_{0}=(\dot{\varphi }-{\dot{\varphi }}_{0})/\cos\vartheta_{0}=\frac{M_{t}}{S_{z}\cos\vartheta_{0}}[1+(S_{z}/S_{y}-1)\sin^{2}\vartheta_{0}]$$

The equivalent torsional stiffness is defined as $\tilde{T}=M_{t}/\left(\varphi^{\prime }-\varphi_{0}^{\prime }\right)$. From Equation (33), the equivalent torsional stiffness of the helical strand can be expressed as

(34) $$\tilde{T}=S_{z}\cos\vartheta_{0}{\left[1+(S_{z}/S_{y}-1)\sin^{2}\vartheta_{0}\right]}^{-1}$$

#### 3.4.2. Equivalent Bending Stiffness of the Helical Strand

**Figure 6:**
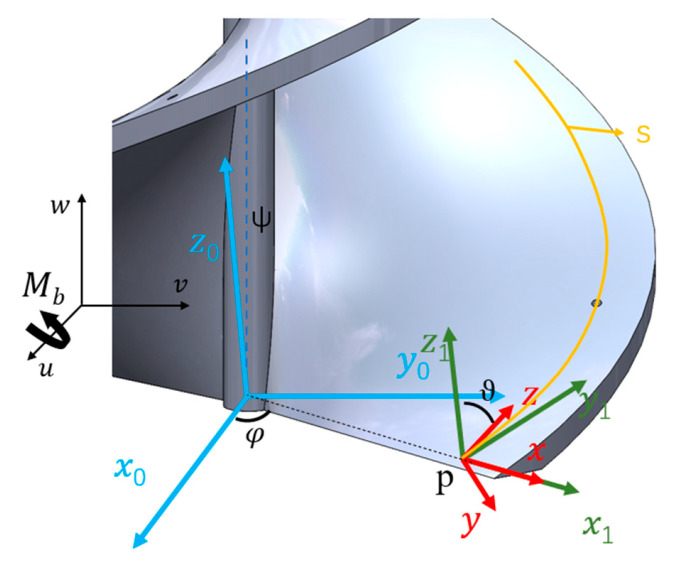
Reference frame of the helical continuum unit under the bending moment.

We assume that the helical strand bends under a pure bending moment and approximate its deformed curve by a constant-curvature model. Figure 6 shows the bending configuration of a single helix. We attach a base coordinate frame $\left\{o,\;u,\;v,\;w\right\}$ to the center of the curve. The *u*-axis is parallel to the bending moment *M_b_*. The *w*-axis is parallel to the central axis of the helical continuum unit in the undeformed state. We then translate the base coordinate frame to point *o*_0_ and rotate it about the *u*-axis by an angle *ψ*, which gives the coordinate frame $\left\{o_{0},\;x_{0},\;y_{0},\;z_{0}\right\}$.

We construct the body-fixed coordinate frame in a manner similar to that described above. First, we translate the base coordinate frame to point *p* and rotate it about the *z*-axis by an angular variable *φ*, which gives the coordinate frame $\left\{p_{1},\;x_{1},\;y_{1},\;z_{1}\right\}$. Next, we rotate $\left\{p_{1},\;x_{1},\;y_{1},\;z_{1}\right\}$ about the *x_1_*-axis by an angle *ϑ*, which gives the body-fixed coordinate frame $\left\{p,\;x,\;y,\;z\right\}$. When the helix undergoes bending deformation, the projections of the deformation onto the *x*-axis, *y*-axis and *z*-axis can be expressed as

(35) $$\begin{array}{l} \kappa_{x}=\dot{\vartheta }+\dot{\psi }\cos\vartheta \\ \kappa_{y}=-\dot{\varphi }\sin\vartheta -\dot{\psi }\cos\vartheta \sin\varphi \\ \tau_{z}=\dot{\varphi }\cos\vartheta -\dot{\psi }\cos\vartheta \sin\varphi \end{array}$$

The corresponding projections of the moment onto the cross-section at point *p* in the body-fixed coordinate frame can be derived as

(36) $$\begin{array}{l} M_{x}=S_{x}(\kappa_{x}-\kappa_{x0})\\ M_{y}=S_{y}(\kappa_{y}-\kappa_{y0})\\ M_{z}=S_{z}(\kappa_{z}-\tau_{z0}) \end{array}$$

where *S_x_* denotes the bending stiffness of the helix about the *x*-axis. In the undeformed state of the helical chain, *ϑ* and *ψ* remain constant. Therefore, ${\dot{\vartheta }}_{0}=0$ and ${\dot{\psi }}_{0}=0$. The projection of the bending moment *M_b_* onto the axes of the coordinate frame $\left\{p,\;x,\;y,\;z\right\}$ can be expressed as

(37) $$\begin{array}{l} M_{x}=M_{b}\cos\varphi \\ M_{y}=-M_{b}\sin\varphi \cos\vartheta \\ M_{z}=-M_{b}\sin\varphi \sin\vartheta \end{array}$$

From Equations (36) and (37), we obtain

(38) $$\begin{array}{l} S_{x}(\dot{\vartheta }+\dot{\psi }\cos\vartheta )-M_{b}\cos\varphi =0\\ S_{y}(-\dot{\varphi }\sin\vartheta -\dot{\psi }\cos\vartheta \sin\varphi )+M_{b}\sin\varphi \cos\vartheta =0\\ S_{z}(\dot{\varphi }\cos\vartheta -\dot{\psi }\cos\vartheta \sin\varphi )+M_{b}\sin\varphi \sin\vartheta =0 \end{array}$$

According to [21], the equation can be derived as

(39) $$\dot{\psi }=\frac{M_{b}}{S_{x}}[1+\frac{1}{2}(S_{z}/S_{y}-1)\sin^{2}\vartheta_{0}]$$

The equivalent bending stiffness is defined as ${\tilde{S}}_{b}=M_{b}/\psi^{\prime }$, where $\psi^{\prime }=\dot{\psi }/\cos\vartheta$. The equivalent bending stiffness of the helical strand can therefore be derived as

(40) $$\tilde{B}=2\cos\vartheta_{0}{\left[\frac{1}{S_{x}}+\frac{1}{S_{y}}\left(1+\left(\frac{S_{y}}{S_{z}}-1\right)\sin^{2}\vartheta_{0}\right)\right]}^{-1}$$

#### 3.4.3. Equivalent Stiffness of the MOSR

The stiffness of the MOSR is determined by the combined stiffness of the two helical strands. Equations (34) and (40) give the equivalent torsional stiffness and equivalent bending stiffness of a single helix, respectively. We can therefore express the equivalent stiffness of the MOSR as

(41) $$\begin{array}{l} T_{c}=\lambda tn\tilde{T}\\ B_{c}=\lambda bn\tilde{B} \end{array}$$

Since the theoretical bending stiffness is established under ideal assumptions, it cannot fully capture the effects of manufacturing tolerances, assembly clearance, material nonlinearity, and contact deformation in the physical prototype. Therefore, a compensation coefficient *λ_b_* is introduced to account for the cumulative influence of these unmodeled factors. Physically, *λ_b_* represents the ratio between the experimentally identified equivalent bending stiffness and the theoretical bending stiffness, thereby correcting the ideal analytical model to better match actual structural behavior. Similarly, the torsional compensation coefficient *λ_t_* is introduced to compensate for the discrepancy between the theoretical torsional stiffness and the experimentally observed response caused by structural compliance, assembly errors, and nonlinear deformation. The values of *λ_b_* and *λ_t_* were determined by calibrating the analytical model against the experimental stiffness measurements reported in reference [22], and *n* denotes the number of helical strands. We can then derive the equivalent bending stiffness *B_c_* and torsional stiffness *T_c_* of the MOSR. The bending and torsional deformations of the structure can be calculated as

(42) $$\begin{array}{l} \theta_{b}=\frac{M_{b}l}{B_{c}}\\ \theta_{t}=\frac{M_{t}l}{T_{c}} \end{array}$$

### 3.5. Tendon-Driven Kinematic Analysis of the Helical Continuum Unit

To simplify the analytical derivation while preserving the dominant deformation characteristics of the proposed MOSR, the tendon-driven helical continuum unit is modeled under the constant-curvature assumption. This assumption considers that the continuum segment bends with uniform curvature along its centerline, while axial extension, tendon friction, and local structural deformation are neglected. Such simplifications have been widely adopted in the preliminary kinematic modeling of tendon-driven continuum robots because they provide an efficient analytical solution for motion planning and workspace prediction.

**Figure 7:**
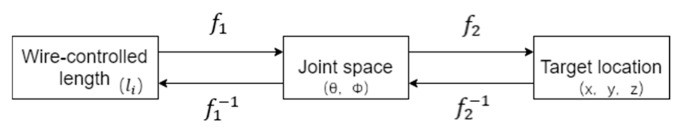
Kinematic model of the MOSR.

As shown in Figure 7, the kinematic model consists of two parts. The first part describes the relationship between changes in tendon length and the joint-space variables. The second part establishes a mapping from the joint space to the position and orientation of the head of the MOSR in three-dimensional space.

In the structural design, the helical continuum unit contains multiple through-holes for tendon routing. By independently controlling the length of each tendon, we can precisely regulate the bending shape of the unit and the pose of its end effector.

**Figure 8:**
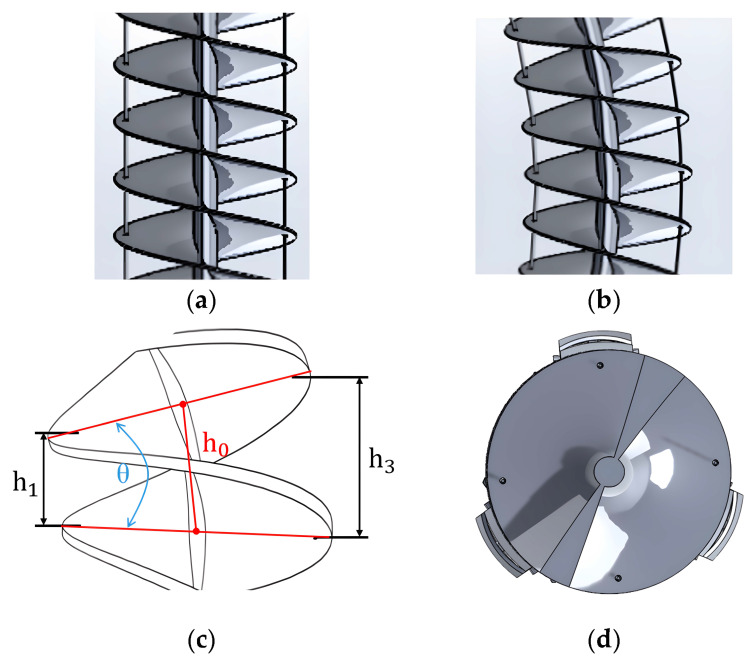
Bending analysis of the MOSR: (**a**) resting configuration section diagram; (**b**) bending configuration section diagram; (**c**) bending analysis diagram; (**d**) cross-sectional view at rest.

The bending analysis of the MOSR is illustrated in Figure 8. Figure 8a and Figure 8b show the cross-sectional configurations of the MOSR in the undeformed and bent states, respectively. Figure 8c and Figure 8d, respectively, show the bending analysis diagram and the cross-sectional diagram at the static position. We can simplify the helical continuum unit as a serial structure with *N* pitches. In the figure, *d_t_* denotes the distance between two driving tendons; *l_1_*, *l_2_*, *l_3_*, and *l_4_* denote the tendon lengths between two adjacent disks during steering; and *h_0_* denotes the gap between two adjacent disks along the centerline. If we neglect the axial deformation of the MOSR, this gap remains constant. The tendon length in the undeformed state is denoted by *l_0_*. After the MOSR bends, the tendon length variation can be expressed as

(43) $$\begin{array}{l} l_{1}\approx N(h_{0}-\frac{\theta d_{t}}{2}\cos\phi )=l_{0}-\frac{N\theta d_{t}}{2}\cos\phi \\ l_{2}\approx N(h_{0}-\frac{\theta d_{t}}{2}\sin\phi )=l_{0}-\frac{N\theta d_{t}}{2}\sin\phi \\ l_{3}\approx N(h_{0}+\frac{\theta d_{t}}{2}\cos\phi )=l_{0}+\frac{N\theta d_{t}}{2}\cos\phi \\ l_{4}\approx N(h_{0}+\frac{\theta d_{t}}{2}\sin\phi )=l_{0}+\frac{N\theta d_{t}}{2}\sin\phi \end{array}$$

where *n* denotes the number of helical strands, *l* denotes the length of the flexible helical segment, and *p* denotes the pitch of the helical strand, with *N = nl/p*. Based on the bending angle *θ* and bending direction *ϕ*, we can derive the mapping relationship between the tip position and the intermediate nodes as

(44) $$\begin{array}{l} x=\sin\theta l_{0}/\theta \\ y=\sin\phi (1-\cos\theta )l_{0}/\theta \\ z=\sin\theta l_{0}/\theta \end{array}$$

The above equations can be used to calculate the tip position and provide a theoretical basis for tendon-length planning and end-effector control of the MOSR.

It should be noted that the above kinematic formulation is derived under the ideal constant-curvature assumption. During practical operation, the driving tendons slide through multiple guide holes distributed along the helical continuum unit. As the bending angle increases, friction between the tendon and the guide holes gradually accumulates, resulting in non-uniform tendon tension transmission. Consequently, the actual curvature distribution deviates from the ideal constant-curvature profile, especially under large bending deformation or external loading. Therefore, the proposed model is mainly intended for preliminary kinematic prediction and motion planning rather than high-precision deformation estimation.

## 4. Experimental Setup

### 4.1. Modeling and Simulation Setup

To evaluate the effectiveness of the proposed equivalent stiffness model, finite element analyses were conducted in ANSYS Workbench (Version Number: 2022 R1). The helical continuum unit was modeled with Polyamide 1010 material properties (Young’s modulus 1500 MPa, Poisson’s ratio 0.38). Torsional moments and bending moments were separately applied to the biomimetic snake structure in ANSYS, and the numerical results were compared with the values predicted by Equation (42).

To analyze the dynamic load characteristics of the gripper during actual movement, a 1 s motion trajectory of the gripper from opening to closing was designed based on a fifth-order polynomial trajectory planning method. The trajectory was then substituted into the established Lagrange dynamics equations. To reveal the torque coupling characteristics of the three-joint linkage mechanism across the entire posture space, a simulation analysis was conducted on the variation patterns of joint torques with respect to joint angles.

Based on the kinematic model analysis in Chapter 3, we were able to simulate and obtain the reachable workspace of the fingers. For a single finger, its workspace can be approximately regarded as a two-dimensional plane perpendicular to the rotating joint axis. Three fingers combined form a Gripper. The spherical docking Gripper thus formed is used to grasp small or fragile objects. It is also subject to constraints imposed by the lengths of the connecting rods and the overall size of the mechanism. Therefore, we limited the rotation ranges of the proximal joint (PJ), middle joint (MJ), and distal gripper Segment (DJ) linked joints to relatively small intervals, namely 0–7°, −10–5°, and −13–10°. These restrictions ensure grasping accuracy, avoid collisions between the fingertips, reduce actuator load, and improve the operation response speed. The D-H parameters and their physical meanings are shown in Table 1 below. Here, $l_{1}$ = 38 mm, $l_{2}$ = 23.5 mm, $l_{3}$ = 25.5 mm

### 4.2. Modular and Multi-Module Performance Test Setup

**Figure 9:**
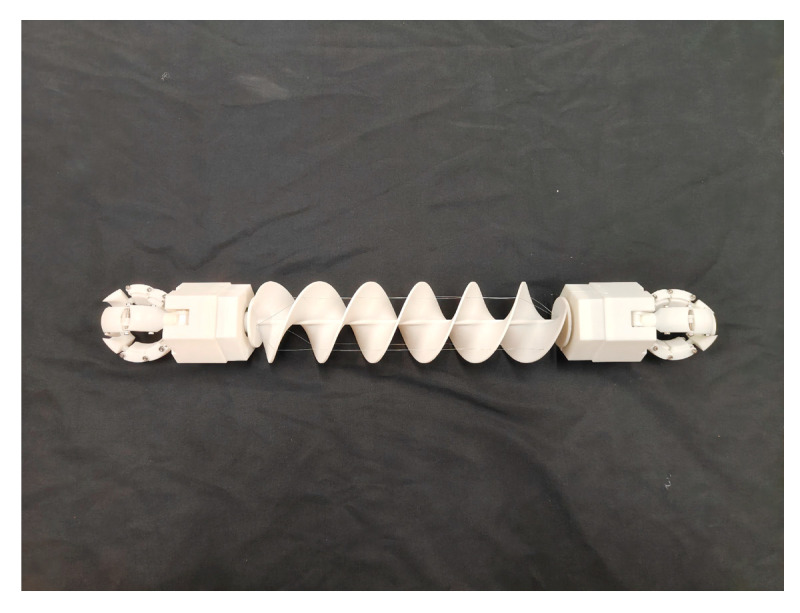
MOSR prototype.

A biomimetic snake prototype with a complete helical continuum unit was fabricated using 3D printing technology, as shown in Figure 9. The material used in this study is polyamide 1010. Its Young’s modulus is 1500 MPa, and its Poisson’s ratio is 0.38.

Under the actuation of the driving tendons, this unit can undergo continuous bending, which enables planar steering and spatial posture adjustment of the robot. When encountering lateral obstacles, the MOSR can avoid them through leftward and rightward bending in the horizontal plane. When facing obstacles that are difficult to bypass, such as steps and stones, the MOSR can also bend in the vertical plane to perform a head-lifting motion, thereby enhancing the obstacle-crossing capability of its front end. In order to test the kinematic model of the spiral continuous unit and verify the accuracy of Formula (44), one end of the unit was fixed to the base, and loads of 0 N, 5 N, and 10 N were applied at the other end. Lateral displacement profiles along the unit’s length were extracted from high-resolution digital photographs and analyzed with ImageJ (Version 1.54f). Considering that the repeated movements of the driving tendons might cause wrinkles and interfere with the experimental results, three experiments were repeated under the same conditions, and the average deformation of the MOSR prototype was recorded.

In addition to conducting simulations, the spherical docking gripper also needs to have its grasping performance tested and simultaneously monitor the angle during the grasping process. The test subjects included regular shapes and irregular shapes. The grasping mode was recorded as “enveloping grasping” (where all finger joints were in contact with the object) or “fingertip grasping” (where only the distal parts were involved). The grasping stability was qualitatively evaluated by observing whether the object remained in place under gentle shaking by hand.

After obtaining the docking angle through simulation experiments, based on the relative position relationship of the two modules, docking experiments can be divided into two situations: straight docking and oblique docking. Straight docking refers to the situation where the central axes of the helical structures of the two modules are on the same straight line, and the modules approach each other at a speed of approximately 5 mm per second to complete the docking. This operating condition is mainly used to verify the docking capability of the all-round docking mechanism in the axial alignment situation, as well as the comprehensive movement characteristics of the combined modules after docking. In contrast, oblique docking refers to the situation where the central axes of the helical structures of the two modules maintain a certain angle (rather than being on the same straight line), and the docking is completed within the docking angle range allowed by the all-round docking mechanism. This operating condition is closer to the actual application situation in complex environments, because the modules often cannot maintain strict coaxial alignment throughout the docking process. If the two modules maintain a mechanical connection and no relative rotation or separation under gentle manual pulling (about 2 Newtons), it is considered that the docking is successful.

The same MOSR prototype was used for repeated tests of gripping, docking, and deformation. For the four core tests—gripping performance, attitude angle detection, straight/tilted docking, and bending deformation—each test was independently repeated 10 times, with a sample size (n = 10) to ensure the validity of the statistical analysis. All tests were conducted under the same environment, control parameters, and assembly conditions to eliminate external interference.

## 5. Results

### 5.1. Helical Continuum Unit

#### 5.1.1. Finite Element Validation of the Stiffness Model

**Figure 10:**
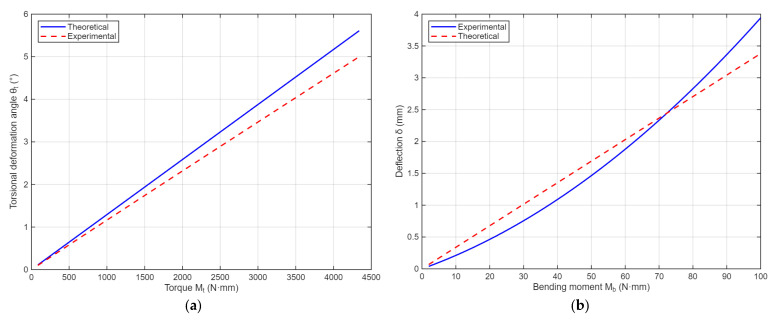
Equivalent stiffness verification: (**a**) torque and torsional deformation angle; (**b**) moment and bending deformation quantity.

As shown in Figure 10, the results show that, under both torsional and bending loading conditions, the theoretical and finite element curves increased monotonically with deformation and exhibited consistent overall trends, indicating that the proposed model could adequately capture the fundamental mechanical response of the double-helical biomimetic snake structure. With the correction factors *λ_t_* = 1.2 and *λ_b_* = 0.9, good agreement was obtained in the torsional case, where the mean relative error was approximately 11.57%, and the maximum absolute error was 0.6. The error distribution remained relatively concentrated, suggesting that the model provided reliable predictions of torsional behavior. In the bending case, the theoretical and finite element results also show good agreement in the small- and moderate-deformation ranges. The mean relative error of the bending deformation was approximately 17.95%, and the maximum absolute error was 1.2 mm. This study focuses on the prototype and preliminary engineering modeling of a biomimetic helical continuum robot. Referring to the application standards for similar equivalent stiffness models in the field, an average relative error of less than 20% in the principle verification stage is sufficient to meet the requirements of preliminary engineering analysis; therefore, this error is within an acceptable range. Based on experimental conditions, the prototype’s maximum effective bending displacement reaches 71 mm, and the calculated maximum absolute error accounts for only 1.74% of the total deformation range, having a minimal impact on the robot’s posture control and operational performance.

#### 5.1.2. Kinematic Model Validation

**Figure 11:**
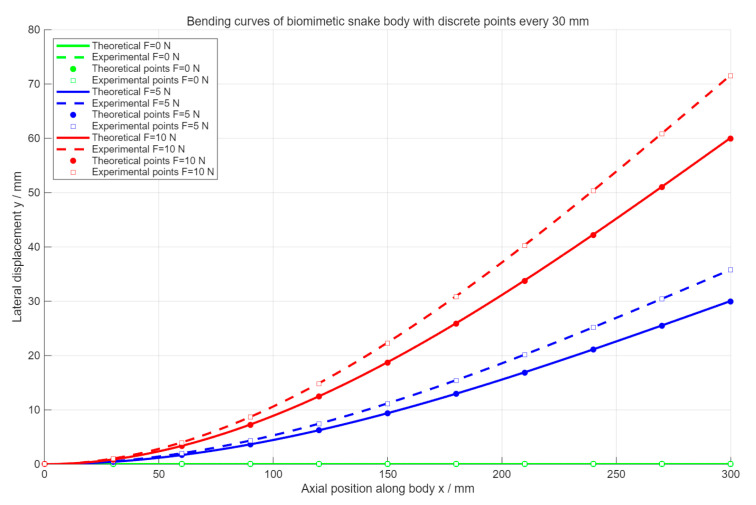
Average deformation of the MOSR.

As shown in Figure 11, when the load increased to 5 N, the theoretical and experimental curves still exhibited the same overall variation trend. Near the fixed end, the difference between the two curves was small. In the middle and rear sections, however, the lateral displacement given by the experimental curve was noticeably greater than the theoretical value.

As can be approximately observed from the free-end position in the figure, the theoretical value was about 30 mm, whereas the experimental value was about 36 mm, yielding a difference of approximately 6 mm, and the error of the difference relative to the length of MOSR was 2%. When the load was further increased to 10 N, this discrepancy became more pronounced. The theoretical and experimental curves still maintained the same bending direction and similar curve profiles, but the experimental curve shifted further upward relative to the theoretical curve, and the deviation near the free end increased significantly. The figure shows that at the free end, the theoretical lateral displacement was about 60 mm, whereas the experimental lateral displacement was about 71 mm, corresponding to a difference of approximately 11 mm, and the error of the difference relative to the length of MOSR was 3.6%. In addition, near the middle section of the body, the experimental values were generally higher than the theoretical values.

#### 5.1.3. Bending Performance Test of the Helical Continuum Unit

**Figure 12:**
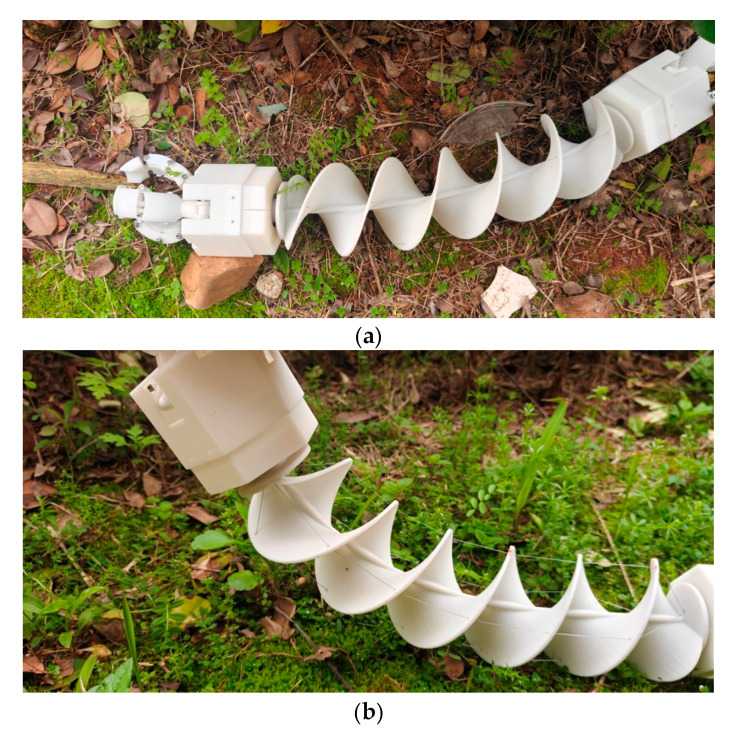
Bending of the MOSR prototype: (**a**) MOSR performs a curved obstacle avoidance movement; (**b**) MOSR performs upward obstacle avoidance movement.

By leveraging the bending deformation capability of the aforementioned MOSR, when driven by the driving link, this unit could undergo continuous bending. This enabled the robot to achieve planar turning and spatial posture adjustment. When encountering lateral obstacles, the MOSR could avoid them through leftward and rightward bending in the horizontal plane, as shown in Figure 12a. When facing obstacles that are difficult to bypass, such as steps and stones, the MOSR could also bend in the vertical plane to perform a head-lifting motion, thereby enhancing the obstacle-crossing capability of its front end, as shown in Figure 12b.

### 5.2. The Grasping Performance of the Spherical Docking Gripper

#### 5.2.1. Workspace Analysis of the Gripper

**Figure 13:**
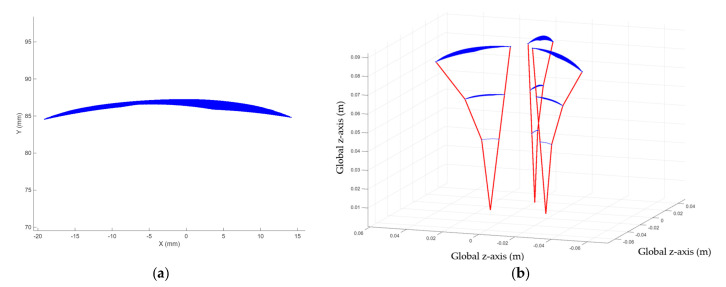
Workspace analysis: (**a**) the fingertip working area under restricted conditions; (**b**) the working area of the spherical docking gripper under restricted conditions.

The simulation results show the fingertip workspace in Figure 13a and the combined workspace distribution of the three fingers of the spherical docking gripper in Figure 13b. The simulation results showed that the fingertip workspace of the proposed underactuated gripper presented a continuous fan-shaped distribution in the plane perpendicular to the proximal joint axis. The maximum reachable height of a single finger was approximately 87 mm, with a lateral span of ±15 mm in the *X* direction, forming a smooth, compact boundary without discontinuous gaps. In the three-dimensional view, the workspace of each finger formed a conical envelope extending from the base joint to the fingertip. The radial boundary values of the section were extracted from several typical axial heights along the Z-axis: at the base location with an axial height *Z* = 45 mm, the minimum radial radius was 4.5 mm and the maximum radial radius was 8.5 mm; at an axial height z = 70 mm, the minimum radial radius was 7.0 mm and the maximum radial radius was 12.0 mm; at the fingertip’s maximum extension height *Z* = 87 mm, the minimum radial radius was 9.5 mm and the maximum radial radius was 15.0 mm. As the axial height increases, both the minimum and maximum achievable radial radii of the section gradually increase.

When multiple fingers were assembled into the gripper, their workspaces exhibited a symmetric arrangement around the central axis, creating an overlapping and continuous reachable region. This symmetric and compact workspace distribution provides a solid foundation for the gripper to perform stable, repeatable enveloping grasping tasks on objects of varying sizes and shapes.

#### 5.2.2. Static Analysis of Underactuated Gripper

**Figure 14:**
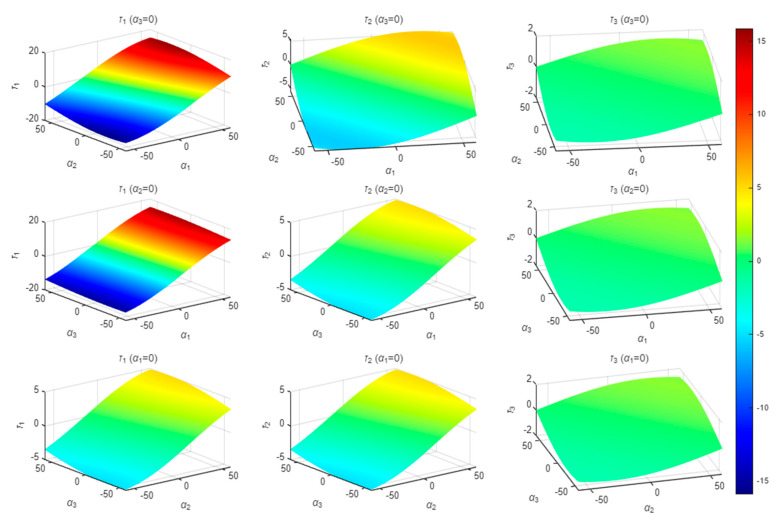
Static moment distribution cloud map.

In Figure 14 below, the X and Y axes represent the rotation angles of the two joints, while the Z axis and the color jointly indicate the magnitude of the static torque required for the corresponding joints, with the unit being N·mm. The rotation angles of the joints are uniformly within the range of $\alpha_{1}$, $\alpha_{2}$, $\alpha_{3}$ ∈ [−60°, 60°], and the static torque is calculated by taking the partial derivative of the gravitational potential energy with respect to the joint rotation angles.

A comparative analysis of the static moment surfaces was conducted. As shown in Figure 14, the torque amplitude and surface shape of each joint exhibit stable gradient differences. The first joint ($\tau_{1}$) has the largest torque variation range and the strongest surface nonlinearity. The torque amplitude of the second joint ($\tau_{2}$) drops significantly, while the torque fluctuation of the third joint ($\tau_{3}$) is minimal, and the surface is almost flat. Under the constraints of $\alpha_{2}=0$ and $\alpha_{2}=0$, the $\tau_{1}$ surface exhibits a typical saddle-shaped strong nonlinear distribution, completely covering the entire color mark range, with torque ranges of [−18.2, 17.6] N·mm and [−17.9, 17.3] N·mm, respectively; the $\tau_{2}$ surface has a unidirectional, gently gradation shape, occupying only a portion of the color mark range, with torque ranges of [−5.3, 4.8] N·mm and [−5.1, 4.9] N·mm, respectively; the τ3 surface is nearly planar, with colors uniformly concentrated within a narrow range, and has the lowest torque amplitude, with ranges of [−1.1, 0.9] NMM and [−1.0, 0.8] NMM, respectively. When the constraint $\alpha_{1}=0$ is applied, the nonlinearity of the $\tau_{1}$ surface is significantly reduced, the color mark coverage area narrows, and the torque range shrinks to [−12.5, 11.8] N·mm, while the global torque does not drop to zero. Under this condition, the surface morphology and color distribution of $\tau_{2}$ and $\tau_{3}$ show no significant changes, and the torque ranges are [−4.9, 4.7] N·mm and [−0.9, 0.8] N·mm, respectively, which are basically consistent with the data of the first two constraint conditions. Overall, the torque amplitudes of the three joints always satisfy the relationship $\tau_{1}>\tau_{2}>\tau_{3}$, with only $\tau_{1}$ showing significant numerical fluctuations due to changes in the constraint conditions. The torque characteristics of $\tau_{2}$ and $\tau_{3}$ show good consistency.

#### 5.2.3. Torque Characteristic Analysist

**Figure 15:**
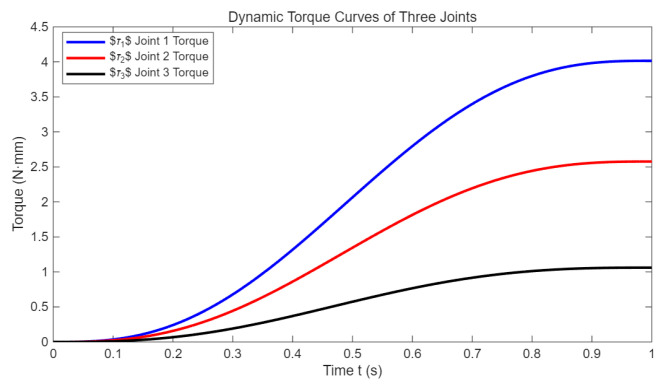
Dynamic torque curve.

Figure 15 shows the curves of the dynamic torque of each joint over time. The curves indicate that the joint torque during movement is influenced by both gravity and dynamic inertia, exhibiting a pattern of “slow start—linear growth—smooth finish.” Since the initial posture is close to the gravity self-balancing position, there is no significant reverse torque during the start-up phase, and the torque slowly increases from near zero. During movement, the torque increases approximately linearly with the posture change. The peak torque values for each joint are approximately 4.0 N·mm for $\tau_{1}$, 2.6 N·mm for $\tau_{2}$, and 1.1 N·mm for $\tau_{3}$. The earlier the joint number, the greater the torque requirement, verifying the theoretical analysis that the base joint is the main load-bearing joint. In the later stages of movement, the gripper reaches the target posture, the dynamic load disappears, and the torque tends to stabilize.

#### 5.2.4. Angle Monitoring Experiment

**Figure 16:**
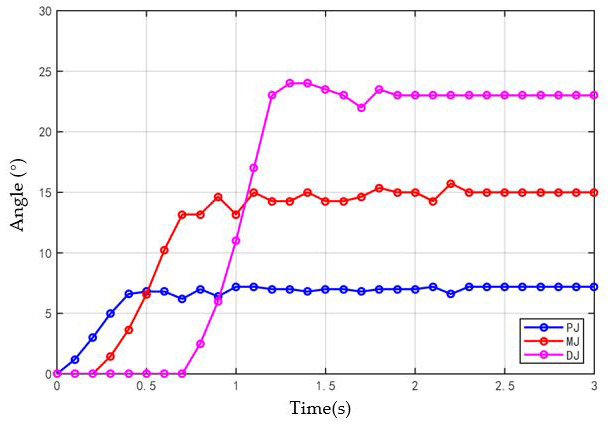
Changes in the angle of the grasping joint.

The accuracy of sensor data plays a decisive role in the result analysis. After installing and confirming the angle sensor is fixed and connecting the circuit, angle detection during the grasping process can be achieved. Figure 16 shows the angle change of the gripper during the grasping process. As can be seen from Figure 16, the angle change fluctuates significantly during grasping, stabilizing sequentially from the proximal end to the distal end of the gripper. Due to the large output torque of the motor, it only takes about 1 s from start to stable grasping, with link III (DJ) taking slightly longer to stabilize. It can be seen that the final bending angle during grasping stabilizes approximately at PJ $\theta_{1}=7{}^{\circ}$, MJ $\theta_{2}=15{}^{\circ}$, and DJ joint $\theta_{3}=23{}^{\circ}$.

#### 5.2.5. Grasping Performance Test

**Figure 17:**
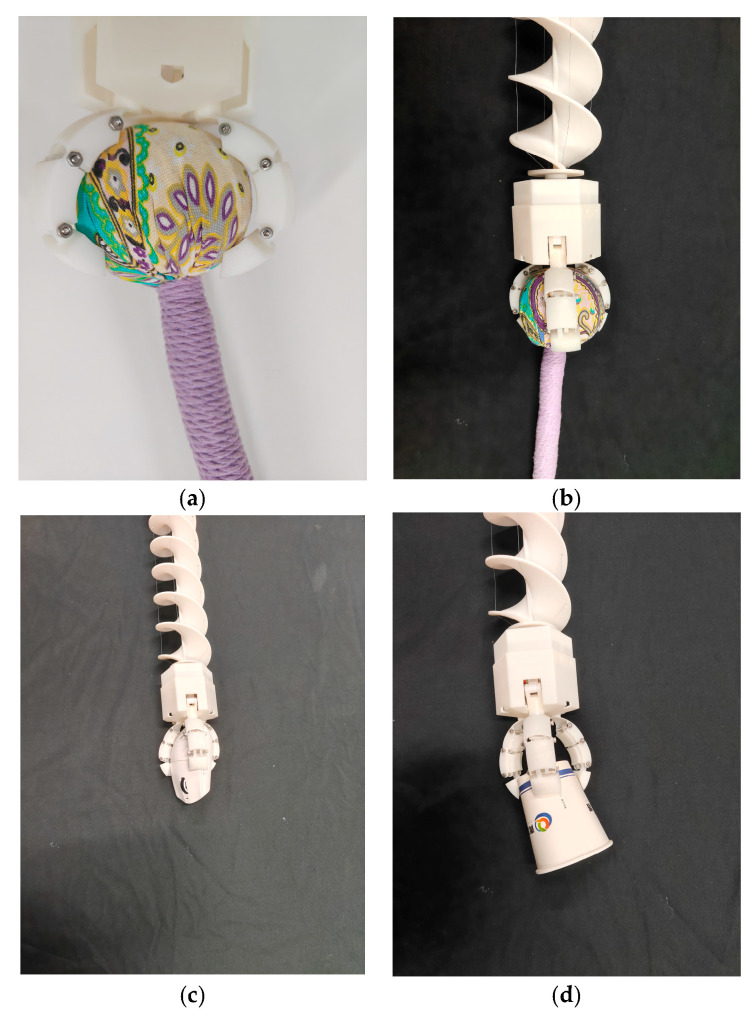
Grasping test of the terminal spherical docking gripper: panels (**a**), (**b**), and (**c**) represent the enveloping grasping mode; panel (**d**) represents the fingertip grasping mode.

The aforementioned gripper workspace provided a theoretical basis for the grasping experiment. Figure 13 presents the experimental results of the terminal spherical docking gripper. The gripper demonstrated the ability to grasp objects of different shapes and sizes, highlighting its versatility beyond its primary function as a modular connector. Figure 17a–c illustrates enveloping grasping. When the target object was relatively large in size and mass, all three segments participated in the grasping process, which also represented a passive adaptive grasping mode.

During grasping, the finger first underwent coupled motion, and Internal Contact Plate I was the first component to contact the object surface. After contact occurred, the contact plate no longer followed its initial trajectory completely, but instead underwent relative displacement along the alignment direction under the guidance of the finger shell. This displacement was transmitted to the linkage mechanism through the pin in the guide rail, driving Link II to rotate about the joint until the object was fully enveloped. It could be observed that each underactuated finger conforms closely to the object surface, which enabled stable grasping. Figure 17d shows a grasping mode in which only the fingertip of the underactuated finger was used to grasp the object. In this mode, the object was not fully enveloped by the spherical docking gripper, and the grasping stability was lower than that of the enveloping grasping mode. To quantitatively evaluate the grasping performance, each grasping task was repeated 10 times under identical experimental conditions. All the test subjects successfully completed the grasping task, and the overall success rate of grasping was 85%. These results demonstrate the excellent repeatability and adaptability of the proposed underactuated gripper for objects with different geometries. Nevertheless, this mode was suitable for grasping objects of moderate size and low mass.

### 5.3. The Docking Performance of the Spherical Fixture

#### 5.3.1. Feasible Docking Angle Analysis

By substituting the model parameters in Table 2 into Equations (8)–(11), we could determine the feasible docking range of this mechanism.

**Figure 18:**
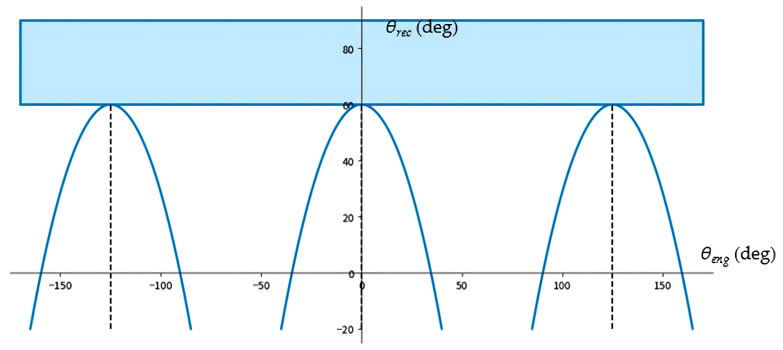
Feasible docking region on the receiver surface at an opening distance of 5.7 mm.

As shown in Figure 18, when the opening distance of the engager was 5.7 mm, 61.1% of the receiver surface lay within the feasible docking region. The same docking-angle analysis also applies to docking among more than two modules.

#### 5.3.2. Multi-Module Docking and Reconfiguration

**Figure 19:**
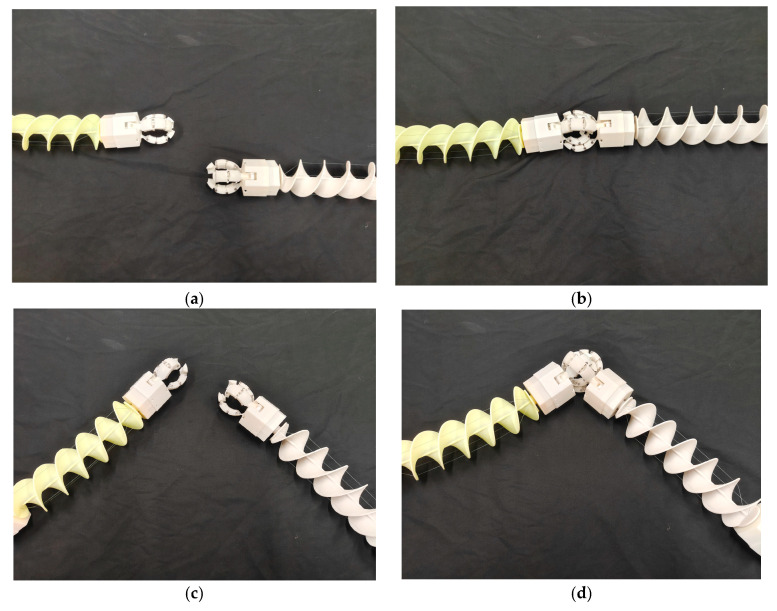
Docking test of the omnidirectional docking mechanism: (**a**) state before straight-line connection; (**b**) state after straight-line connection; (**c**) state before oblique connection; (**d**) state after oblique connection.

Within the aforementioned feasible docking range, multiple docking experiments were conducted on the MOSR. Figure 19 presents the representative results of the multi-module docking experiments. Figure 19a,b correspond to the straight docking condition, where Figure 19a shows the initial state before docking and Figure 19b shows the state after docking is completed. It could be seen that under this condition, the two modules could successfully engage along a common axis and form a continuous overall configuration after connection. Figure 19c,d correspond to the oblique docking condition, where Figure 19c shows the state before docking and Figure 19d shows the state after docking is completed. The experimental results indicated that even when a certain initial angle exists between the two modules, stable docking could still be achieved as long as this angle lay within the allowable working range of the omnidirectional docking mechanism, while the structural integrity of the connected system was maintained after docking. After the two modules are stably docked, the overall structure still retains the bending ability of a continuum, enabling small-scale attitude adjustments and local obstacle avoidance, proving that the docking structure does not completely restrict the original degrees of freedom of motion of the individual units. To further evaluate the reliability of the proposed omnidirectional docking mechanism, each docking mode was repeated 10 times under identical experimental conditions. The experimental results showed that all straight docking and oblique docking trials were completed successfully, corresponding to a docking success rate of 100%. These statistical results further verify the repeatability and robustness of the proposed docking mechanism.

### 5.4. Performance Test of the Helical Continuum Unit (Forward Thrust and Speed)

The magnitude of the thrust is related to both the design parameters of the helical continuum unit and the physical properties of the ground surface. The forces exerted by the ground on the blade mainly include the thrust that drives the locomotion mechanism forward and the friction acting in the direction opposite to the blade motion. In addition, the locomotion mechanism is also subjected to ground resistance during travel. The thrust acts along the normal direction of the blade and can be decomposed into an axial force along the axis of the helical continuum unit and a lateral force in the horizontal direction perpendicular to the axis. Considering an infinitesimal sector element on the helical blade, its area can be expressed as

(45) $$dA\left(\theta \right)=\pi \left(r^{2}-r_{1}^{2}\right)d\theta$$

where *dA* denotes the area of the infinitesimal sector element, and *dθ* denotes the corresponding central angle of the element. Accordingly, the resultant force acting on a single helical blade, denoted by *F*, can be expressed as

(46) $$\left\{\begin{array}{c} F=\int \left(\tau \cos\xi -\sigma \sin\xi \right)dAd\theta \\ \xi =\tan^{-1}\left[\cos\left(2\pi -\eta \right)-\tan\theta \right] \end{array}\right.$$

where *τ* denotes the shear force exerted by the helical blade on the ground, *σ* denotes the normal pressure exerted by the ground on the helical blade, and *θ* denotes the central angle corresponding to the contact surface between a single helical blade and the ground.

The axial force can be expressed as: $F_{y}=\sum \mathrm{sgn}\left(\omega \right)F\cos\eta$

The lateral force can be expressed as: $F_{x}=\sum \mathrm{sgn}\left(\omega \right)F\sin\eta$

where, with the clockwise direction taken as positive, the value of *sgn(ω)* is given by

(47) $$\mathrm{sgn}(\omega )=\left\{\begin{array}{c} 1,\;\omega >0\\ 0,\;\omega =0\\ -1,\;\omega <0 \end{array}\right.$$

where *ω* denotes the rotational speed of the helical continuum unit, *F_y_* denotes the axial force exerted by the ground on the helical continuum unit, and *F_x_* denotes the lateral force exerted by the ground on the helical continuum unit.

**Figure 20:**
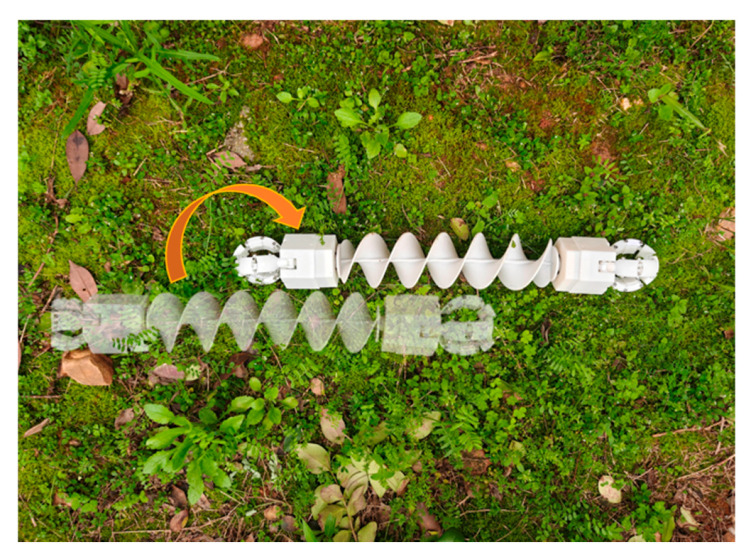
Locomotion experiment of the MOSR prototype on grass.

As shown in Figure 20, a single MOSR module can roll on a grassy surface. The prototype’s travel speed test was conducted on a grassy surface using a fixed-distance timing method, with a test path length of 500 mm. The experiment was repeated 10 times, and the average travel speed of the robot was calculated to be 15.3 mm/s (2 body lengths per minute), with a standard deviation of 0.21 mm/s. The results showed good stability across multiple measurements.

## 6. Discussion

The verification results of the stiffness model indicate that Equations (40)–(42) can effectively predict the equivalent stiffness, bending deformation, and torsional deformation of the double-helical MOSR design. This agreement confirms the reliability and accuracy of the proposed stiffness model for engineering applications.

The kinematic results indicate that under external loading, the actual bending deformation of the prototype was greater than that predicted by the theoretical model, and the model underestimated the overall deformation under load. These results indicate that as the external load increased, the deviation between the theoretical model and the actual response gradually increased, and the predictive capability of the model for bending deformation under higher loads declined. The magnitude of the error was positively correlated with the bending angle of the MOSR. As the bending angle increased, the deviation between the theoretical and measured results became progressively larger. The main reason for this phenomenon is that the kinematic model was derived on the basis of the constant-curvature assumption, whereas the actual prototype was affected by friction between the driving tendon and the guide holes during bending. This effect weakened the uniform bending characteristic of the continuum structure along its length and caused the actual curvature distribution to deviate from the ideal state. As the bending angle increased, the contact interaction between the tendon and the guide holes became stronger, the friction effect became more evident, and the model error increased accordingly. In addition, friction causes progressive attenuation of tendon tension along the transmission path, resulting in a non-uniform curvature distribution rather than the ideal constant-curvature profile assumed in the analytical model. Consequently, the simplified model tends to underestimate bending deformation at relatively large bending angles and under external loads. Nevertheless, the theoretical predictions remain in good agreement with the experimental trends, indicating that the proposed model is still suitable for preliminary kinematic analysis and engineering design. Future work will incorporate friction-compensated tendon transmission and variable-curvature modeling to further improve prediction accuracy.

The differences in static moment distribution cloud maps mainly occur in the proximal joints, which bear the gravitational load of all rear links. The moment is controlled by the pose coupling of multiple links, resulting in a significant coupling effect. The distal joints only bear the weight of their own links; changes in the front joint’s pose cannot alter its center of gravity lever arm relative to its own axis of rotation, leading to a weak coupling moment effect. Ultimately, this causes the moment amplitude of the three joints to decrease progressively along the transmission chain. Setting either $\alpha_{2}$ or $\alpha_{3}$ to zero does not change the inherent mechanical characteristics of the base joint, which involves large loads and strong coupling. Fixing the base joint to zero only compresses the moment fluctuation range but cannot eliminate the gravitational coupling moment caused by the pose deviation of the rear links. This is the fundamental reason why the joint angle is zero, but the moment is not zero. The force states of $\tau_{2}$ and $\tau_{3}$ are determined only by the relative pose of the rear links and are not affected by the proximal constraint conditions. Therefore, the moment distribution patterns of these two joints remain stable under the three working conditions.

The bending performance of the MOSR is jointly influenced by the material properties of the helical continuum unit, the helical structural parameters, and the parameters of the constraint shaft, and its locomotion performance can be optimized by adjusting these key parameters. The results indicate that the helical continuum unit can satisfy the requirements for steering and obstacle crossing under certain conditions and exhibits good locomotion dexterity and environmental adaptability.

In the angle-monitoring experiment, all angles were uniformly taken as positive values for ease of measurement, and the workspace and movement trajectory of the gripper remained unchanged. Experimental results showed that the angle changes in the gripper were all within the predicted range. Although there were significant fluctuations in the initial stabilization phase, the subsequent process became smoother, enabling the gripper to perform its grasping and connecting functions.

Furthermore, the omnidirectional docking mechanism, which permits engagement over 61.1% of the receiver’s spherical surface at an opening distance of 5.7 mm, offers a substantially larger reconfiguration workspace than the unidirectional bayonet connectors commonly used in existing modular robots [1]. The non-docking region mainly results from potential collisions with the neck and the base of the connector.

The straight docking and oblique docking experiments demonstrated the connection capability of MOSR. Straight docking was mainly used to verify the docking capability of the omnidirectional docking mechanism under axial alignment and the integrated locomotion characteristics of the combined modules after docking. Under oblique docking conditions, the engager and the receiver can still establish a stable connection within a certain angular range, which indicates that the omnidirectional docking mechanism has good spatial adaptability. In addition to the qualitative observations, the repeated experiments consistently achieved high success rates in grasping, docking, and obstacle-crossing tests, demonstrating the repeatability and robustness of the proposed MOSR. After docking is completed, either the engager or the receiver can continue to move forward, and a certain degree of bending motion can also be achieved in the connected state. This result shows that the connected modular system is not a static combination, but still retains a certain level of active locomotion capability and can adapt to more complex working environments. Furthermore, within the allowable angular range of the omnidirectional docking mechanism, the oblique docking mode can provide the basis for progressive stacking of multiple MOSR modules, thereby enabling the system to expand from one-to-one docking to one-to-many docking. This characteristic is of particular significance for the modular serpentine robotic system, because it not only supports the basic docking function, but also provides a structural foundation for subsequent multi-module reconfiguration, morphological extension, and task switching.

Table 3 compares the comprehensive performance indicators of MOSR with those of other references. In terms of movement speed, the measured speed of the prototype in this paper is 15.3 mm/s. The speed of the rope-driven robot in reference [23] is 27.6 mm/s, which is higher because it does not integrate grasping and docking functions, and the overall weight and load are smaller. Reference [11] does not disclose the movement speed parameters, so this dimension will not be compared further. In terms of core modular reconfiguration capability, reference [23] is a single robot and does not design a module docking interface. Reference [11] adopts a traditional one-way bayonet docking structure, which can only achieve strict coaxial docking and cannot be reassembled at multiple angles. Traditional connectors also have the problem of a limited reconfiguration range. The feasible docking area of the omnidirectional spherical docking mechanism in this paper reaches 61.1%, which can be compatible with direct docking and multi-angle oblique docking, and the module reconfiguration flexibility is significantly improved. In summary, this prototype adds an omnidirectional modular docking and grasping integrated function on the basis of maintaining the same flexible movement capability, which is more suitable for reconfiguration operation scenarios in complex environments.

## 7. Conclusions

This study proposed a MOSR that integrates a DNA-inspired helical continuum unit and an underactuated omnidirectional spherical docking gripper with adaptive fingers into a single module. The results indicate that the proposed design improves modular docking capability, adaptive grasping performance, and the balance between structural stiffness and compliant motion. The helical continuum unit provides two degrees of freedom of bending while maintaining sufficient axial stiffness for tendon-driven actuation. Its equivalent torsional and bending stiffness models show reasonable agreement with the finite element results, with mean relative errors of approximately 11.57% in torsion and 17.95% in bending. The omnidirectional docking mechanism achieves stable straight and oblique docking, and the docking-angle analysis shows that 61.1% of the receiver surface lies within the feasible docking region at an opening distance of 5.7 mm. The adaptive fingers also demonstrate stable grasping of objects with different shapes and sizes. Prototype experiments further verify the feasibility of the design. The robot achieves a locomotion speed of 15.3 mm/s on grass, can traverse grass, cobblestones, concrete pavement, and terrain transitions, and demonstrates planar steering, vertical bending for obstacle crossing, and adaptive grasping. Overall, the proposed system provides a practical mechanical basis for modular bio-inspired serpentine robots operating in confined and unstructured environments. This study focuses on completing the design, performance verification, and functional testing of the two-module docking and reconstruction, laying the foundation for subsequent research on multi-module group collaboration and complex configuration reconstruction.

## Figures and Tables

**Table 1:** The D-H parameter table under constraints and its physical meaning.

i	$\alpha_{i-1}$/°	$a_{i-1}$/mm	$\theta_{i}$/°	$d_{i}$/mm
1	0	$l_{1}$	0~7	0
2	0	$l_{2}$	−10~5	0
3	0	$l_{3}$	−13~10	0

**Table 2:** Parameters of the spherical docking gripper.

Parameter	Symbol	Value (mm)
Central axis radius	$r_{bar}$	10
Width of a single spiral page	$d_{w}$	24.75
Clearance distance	$d_{open}$	5.7
Radius of the sphere	*r*	34

**Table 3:** Quantitative performance comparison of modular serpentine robots.

Research	Locomotion Speed (mm/s)	Docking Configuration and Feasible Range	Actuation Type
MOSR	15.3 (measured on grass)	Omnidirectional spherical docking; 61.1% of the spherical surface is feasible for docking, supporting both straight and multi-angle oblique docking	Tendon-driven (DNA helical skeleton)
Ref. [23] Tendon-Driven Compliant Wheel-Less Snake Robot	Forward: 27.6 Sidewinding: 20.0	Single independent unit, no modular docking and reconfiguration mechanism	Tendon-driven
Ref. [11] Zhao et al. Modular Serpentine Robot	Not disclosed	Unidirectional bayonet docking, only coaxial connection is supported, reconfiguration angle is limited	Servo-driven
Conventional Unidirectional Bayonet Connector	No independent locomotion unit	Rigid unidirectional coaxial docking, extremely limited reconfiguration space	Rigid mechanical connection

## Data Availability

The original contributions presented in this study are included in the article. Further inquiries can be directed to the corresponding author.

## Abbreviations

The following abbreviations are used in this manuscript:

MOSR	Modular Omnidirectional Serpentine Robot
PJ	Proximal Joint
MJ	Middle Joint
DJ	Distal Gripper Segment

## References

[1] Cai Y., Xu H., Wang Y., Chen D., Matusik W., Shou W., Chen Y. (2025). Modular self-reconfigurable continuum robot for general purpose loco-manipulation. IEEE Robot. Autom. Lett..

[2] Chen H., Chen Z., Liu Z., Xiong J., Yan Q., Fei T., Zhao X., Xue F., Zheng H., Lian H. (2025). From coils to crawls: A snake-inspired soft robot for multimodal locomotion and grasping. Nano-Micro Lett..

[3] Zhao M., Nishio T. (2023). Generalized design, modeling and control methodology for a snake-like aerial robot. Sensors.

[4] Cui Y., An X., Lin Z., Guo Z., Liu X.-J., Zhao H. (2024). Design and Implementation of an Underactuated Gripper with Enhanced Shape Adaptability and Lateral Stiffness through Semi-Active Multi-Degree-of-Freedom Endoskeletons. Int. J. Robot. Res..

[5] Onose R., Sawada H. (2024). A Ball-Jointed Tendon-Driven Continuum Robot with Multi-Directional Operability for Grasping Objects. ROBOMECH J..

[6] Merrad A., Amouri A., Cherfia A., Djeffal S. (2023). A Reliable Algorithm for Obtaining All-Inclusive Inverse Kinematics’ Solutions and Redundancy Resolution of Continuum Robots. Arab. J. Sci. Eng..

[7] Hamad F., Fakhouri H.N., Alzghoul F., Zarqou J. (2025). Development and Design of Object Avoider Robot and Object, Path Follower Robot Based on Artificial Intelligence. Arab. J. Sci. Eng..

[8] Cong V.D., Phuong L.H. (2025). Development of a 3D Vision Robot System to Grasp Objects on the Conveyor Using Artificial Intelligence. Arab. J. Sci. Eng..

[9] Le A.B.N., Tran N.T., Dang M.P., Tran H.V., Ho N.L., Dao T.-P. (2025). Design and Kinetostatic Modelling of a New Inspired-Shrimp Constant Force Compliant Mechanism for Robotic Application. Arab. J. Sci. Eng..

[10] Du Y., Zhang S., Zhang Z., Wang H. (2024). Shape deformation analysis and dynamic modeling of a switchable rigid-continuum robot. Robotica.

[11] Zhao N., Zhao S., Zheng T., Qi J., Yang Z., Sui X., Han K., Luo H., Zhou N., Zhao J. (2025). Modular snake-like robot designed for on-site reconfiguration in space exploration. Biomimetics.

[12] Fu Y., Wang W., Zhang H., Li Z. (2023). A Variable-Stiffness Soft Gripper with Low-Energy Mechanical Latching for Robust Grasping. IEEE Robot. Autom. Lett..

[13] Doan H.T.L., Arita H., Tahara K. (2024). Tactile Sensor-Less Fingertip Contact Detection and Force Estimation for Stable Grasping with an Under-Actuated Hand. ROBOMECH J..

[14] Soon R.H., Ren Z., Hu W., Bozuyuk U., Yildiz E., Li M., Sitti M. (2022). On-demand anchoring of wireless soft miniature robots on soft surfaces. Proc. Natl. Acad. Sci. USA.

[15] Hoang S., Karydis K., Brisk P., Grover W.H. (2021). A pneumatic random-access memory for controlling soft robots. PLoS ONE.

[16] Cianchetti M., Ranzani T., Gerboni G., Nanayakkara T., Althoefer K., Dasgupta P., Menciassi A. (2014). Soft robotics technologies to address shortcomings in today’s minimally invasive surgery: The STIFF-FLOP approach. Soft Robot..

[17] Duan J., Zhang K., Qian K., Hao J., Zhang Z., Shi C. (2024). An operating stiffness controller for the medical continuum robot based on impedance control. Cyborg Bionic Syst..

[18] Ryu H.T., Oh S.M., Tae K., Yi B.J. (2022). DNA-helix inspired wire routing in cylindrical structures and its application to flexible surgical devices. Soft Robot..

[19] Spinks G.M., Martino N.D., Naficy S., Shepherd D.J., Foroughi J. (2021). Dual high-stroke and high-work capacity artificial muscles inspired by DNA supercoiling. Sci. Robot..

[20] Ching T., Lee J.Z.W., Win S.K.H., Win L.S.T., Sufiyan D., Lim C.P.X., Nagaraju N., Toh Y.C., Foong S., Hashimoto M. (2024). Crawling, climbing, perching, and flying by FiBa soft robots. Sci. Robot..

[21] Liu Y.Z. (2006). Nonlinear Mechanics of Thin Elastic Rod: Theoretical Basis of Mechanical Model of DNA.

[22] Zhang X., Xian Y.T., Cui Z.W., Chiu P.W.Y., Li Z. (2022). Design and modeling of a novel DNA-inspired helix-based continuum mechanism (DHCM). Mech. Mach. Theory.

[23] Incekara S., Kwon S., Kwon G., Ha J. (2025). Tendon-Driven Compliant Wheel-Less Snake Robot. Adv. Intell. Syst..

